# Reduced-dose Antiarrhythmic Drugs: Valuable or Valueless?

**DOI:** 10.19102/icrm.2020.110404

**Published:** 2020-04-15

**Authors:** James A. Reiffel

**Affiliations:** ^1^Department of Medicine, Division of Cardiology, Electrophysiology Section, Columbia University, New York, NY, USA

**Keywords:** Amiodarone, antiarrhythmic drugs, combination therapy, pharmaceutical therapy, low dose

Amiodarone is usually considered to be the most effective antiarrhythmic drug (AAD) currently available to clinicians. However, we must have a “healthy concern” about its use because of the high potential for adverse toxicity and multiple drug interactions. In this issue of *The Journal of Innovations in Cardiac Rhythm Management*, Chokesuwattanaskul et al.^1^ report on their attempt to assess the safety of low-dose (≤ 200 mg/day) and very-low-dose (≤ 100 mg/day) amiodarone.

Here, I critically address the points made by these authors in hopes of putting them in the proper perspective. In addition, this commentary also discusses the role of low-dose AADs with respect to dose-related differences in pharmacokinetics and pharmacodynamics for some agents as well as the use of combinations of AADs using reduced doses to enhance efficacy and/or to reduce intolerance.

In their conclusion, Chokesuwattanaskul et al.^1^ state that they have demonstrated “the safety of very low-dose amiodarone, which has been prescribed worldwide. In addition, the incidence of side effects from our study is lower than that of a prior meta-analysis, which might encourage physicians to begin or continue to use this useful medication, albeit with limitations as appropriate. Further research should be conducted to provide stronger evidence regarding the relationship between low-dose amiodarone and related side effects.”

However, are these conclusions accurate and, whether or not they are, is there value not only in low-dose amiodarone but also in low doses of other AADs as well?

## Low-dose amiodarone

Chokesuwattanaskul et al.^1^ suggest that low-dose amiodarone is appropriate to consider in the management of cardiac tachyarrhythmias because, in their dataset, it appeared to be safe. However, is their conclusion correct? Moreover, even if it is, the same doses would have to be shown to be effective—which their paper did not address. In other words, a placebo is safe but has no demonstrable efficacy beyond the psychological realm.

The paper by Chokesuwattanaskul et al.^1^ notes that its authors initially identified 2,312 papers but subsequently eliminated almost all of them from consideration, leaving only 10 papers from which to draw their conclusions. One could reasonably question how representative such a low number of papers can be. Moreover, when I looked at the original reports of these 10 papers, I found that some important data appeared to have been missed, while others had only incomplete data, which did not appear to have been considered. Thus, their findings of a pooled estimated incidence of overall side effects for low-dose amiodarone of 17% or the pooled estimated incidence of side effects requiring medication discontinuation of 6% with 200 mg/day of amiodarone and the pooled estimated incidence of overall side effects of 11% or the incidence of side effects requiring medication discontinuation of 2% for the dose of 100 mg/day should be examined further. For example, in the report by Iwasawa et al.,^2^ which included only patients with congenital heart disease, 23% had amiodarone-induced side effects and 10% died. In the study by Takeuchi et al.,^3^ which also included only patients with congenital heart disease, no adverse events other than thyroid issues were reported. Shiga et al.^4^ highlighted in their research a discontinuation rate of 16%, with 13% of study participants having pulmonary changes. Adverse events other than those leading to discontinuation were not discussed. In the paper by Yamada et al.,^5^ there was no information on adverse events except that three of 17 patients had their dose reduced due to sinus bradycardia, liver, or thyroid issues. In the paper by Lee and Tai,^6^ 9.7% discontinued amiodarone, 12% had thyroid dysfunction, and 1.6% experienced hepatic toxicity. In the paper by Gao et al.,^7^ 14% “succumbed to adverse reactions.” Additionally, no specific laboratory data were reported. In the report by Jong et al.,^8^ 6% developed pulmonary fibrosis and 11% discontinued amiodarone (200-mg/day dose). Roy et al.^9^ reported in their study an amiodarone discontinuation rate of 34%, which included nine deaths (six were nonarrhythmic). Other specific adverse effects were not listed. Finally, the paper by McGrew et al.^10^ was only an abstract of low-dose amiodarone-treated patients with atrial fibrillation (AF). Aside from the 12.5% who died during follow-up (but not due to amiodarone, according to the authors), no mention of other adverse events was included in the abstract.

In my view, the abovementioned findings do not seem to support the very low numbers reported by Chokesuwattanaskul et al.^1^ Further, the trials that these authors appeared to have missed also do not support their conclusion. Most striking is the data from the Cardiac Arrest in the Seattle: Conventional Versus Amiodarone Drug Evaluation (CASCADE) study.^11^ In this trial, which compared treatment with amiodarone against treatment with all other antiarrhythmics combined in survivors of out-of-hospital ventricular fibrillation, amiodarone was the most effective. However, more patients stopped taking amiodarone (29%) during follow-up than those who stopped all other “conventional” antiarrhythmics (17%). Even more strikingly, possible amiodarone pulmonary toxicity was diagnosed in 6% at 12 months and 10% at 36 months, with an average maintenance dose of 183 mg/day at 12 months and 185 mg/day at 36 months. Additionally, 22% of the amiodarone-treated patients developed hyperthyroidism or hypothyroidism. Similarly, in the European Myocardial Infarct Amiodarone Trial (EMIAT) trial,^12^ which compared amiodarone to placebo in reducing mortality in patients with left ventricular dysfunction after a recent myocardial infarction, using a dose of 200 mg/day after a prior loading regimen, 38.5% discontinued amiodarone versus 21.4% on placebo, with more patients on amiodarone developing thyroid, pulmonary, or hepatic abnormalities (including three pulmonary toxicity deaths).

That having been said, I applaud the attempts by Chokesuwattanaskul et al.^1^ to undertake an effort to encourage us to use amiodarone in reduced doses when possible. When the use of amiodarone first became widespread and worldwide, its primary rhythm-related indication was for ventricular tachyarrhythmias and its most common maintenance dose was 400 mg/day, following a variable loading regimen. As such, it gained the reputation of being more likely to be effective than any alternative AAD. In the United States, where life-threatening ventricular tachyarrhythmias remain the only approved indication for amiodarone, package inserts (PIs) usually recommend a maintenance dose of 400 mg/day but note some patients may require 600 mg/day, while others may be able to get by with a lesser dosage. The extremely lengthy PIs also contain a very long list of precautions, black-box material, and other dosing concerns and include information about many of the adverse effects being time- and/or total dose-related. In contrast, when used for atrial tachyarrhythmias, such as AF, which is not mentioned anywhere in the PIs and does not have an indication approved by the United States Food and Drug Administration, the typical recommendation for dosing by most authorities is to try up to 200 mg/day, although the Amiodarone: Considerations for Use from the Atrial Fibrillation Toolkit [available from the American College of Cardiology (ACC)] still suggests doses of 200 mg/day to 400 mg/day for AF. Additionally, the 2014 ACC/American Heart Association/Heart Rhythm Society guidelines for the management of AF^13^ state that, “owing to its potential toxicities, amiodarone should only be used after [the] consideration of risks and when other agents have failed or are contraindicated” and suggest that oral doses be administered first as 400 to 600 mg daily in divided doses for two to four weeks, then as 100 to 200 mg once daily for maintenance, whereas the 2016 European Society of Cardiology guidelines^14^ recommend giving “600 mg in divided doses for [four] weeks, 400 mg for [four] weeks, then 200 mg once daily.” None of these guidelines contain data from randomized controlled trials exploring amiodarone versus placebo for AF in a long-term protocol. Accordingly, although it seems advisable for amiodarone to be used in as low a dose as possible for the maintenance of sinus rhythm in patients with AF, the data reported by Chokesuwattanaskul et al.^1^ are not sufficient to support reducing doses below those currently suggested in an evidence-based and, absent efficacy data, incomplete.

## Low-dose issues with other antiarrhythmic drugs

Interesting issues arise when considering low doses of other AADs as well not only for the purpose of avoiding toxicities or other adverse effects, which is always desirable (see below), but also because of differences in a drug’s actions. Here, let us examine propafenone and sotalol as examples of the latter.

Sotalol (d,l-sotalol mixed racemate, as is available clinically)^15–19^ is most commonly considered a class III AAD. It is so because it prolongs the action potential duration (via potassium channel Ik_r_ blockade) and, with it, the QT interval associated with this action in the ventricles. However, sotalol is also a β-blocker and, hence, has class II properties too. Sotalol is almost completely renally excreted. Importantly, the β-blocker actions begin at doses/serum concentrations lower than the class III effects and can be seen with doses beginning at 40 mg twice daily. They also appear to plateau at doses of 240 mg/day in most individuals. In contrast, the class III effects typically begin at doses of 80 mg twice daily (assuming normal renal function) and increase progressively as the dose increases, without a plateau effect. Thus, with sotalol, using the lowest doses essentially facilitate β-blocking action with little or no class III AAD effects. However, with renal impairment, serum concentrations at lower doses are higher than in those patients with normal renal clearance and class III effects will occur at lower-than-usual doses. Accordingly, clinicians who start sotalol dosing at 40 mg twice daily in patients without renal insufficiency are essentially just administering a relatively expensive β-blocker. However, with higher doses, the class III effects will occur—as may a risk of torsades de pointes.^15–19^

Propafenone also has somewhat complex pharmacokinetic and pharmacodynamic dose-related properties.^15,16,20–22^ Propafenone is primarily a class Ic AAD as a result of its potent sodium channel actions. However, it also has weak β-blocking properties and very weak calcium channel–blocking properties. In most individuals—perhaps 90% of patients—propafenone is rapidly metabolized such that its dosing must be three times daily (unless a slow-release preparation is used). However, the rate of metabolism can be overwhelmed by higher doses. As the β-blocking properties reside in the parent compound, the β-blocking effects are not seen in most patients until high enough doses are used. In contrast, in slow metabolizers, β-blocker effects may occur throughout the dosing range. Thus, in patients intolerant of or with contraindications to β-blockers, propafenone’s metabolic pattern may be of clinical significance. Additionally, the metabolism of propafenone tends to change from the acute to the chronic state so that the ratio of metabolites differs between during the first week of treatment and later dates. When slow-release propafenone congeners are used, a greater percentage of the dose is metabolized such that a reduced β-blocking effect is likely, although the total daily dose must be higher than of the immediate-release preparation (as is typical with all slow-release preparations). Accordingly, low-dose propafenone is more likely not to exhibit β-blocking effects in most individuals, whereas high doses may exhibit such, especially with the immediate-release preparation. Thus, the dose and metabolic pattern determine the β-blocker manifestations, which are generally weak, and the variation from patient to patient.^15,16,20–22^

## Combining antiarrhythmics, both administered at a low dose, to maintain efficacy and reduce side effects

Many conditions are refractory to single-drug therapy and require combinations of agents to provide adequate control—consider patients with hypertension, diabetes, and heart failure, for example. Similarly, arrhythmias refractory to single AADs are not uncommon, including AF. Notably, for ventricular tachyarrhythmias, AAD combinations (AAD-Cs) have a long history of use, especially in the era of treatment prior to the availability of implanted defibrillators. However, relatively few reports exist on the subject of AAD-Cs when provided following the failure of AADs used singly or serially for AF.^23^ Importantly, AADs may fail either due to inefficacy or intolerance.

In my practice, I have used AADs in combination over the past several decades for both ventricular and supraventricular arrhythmias.^24^ In a previously performed but, to date, unpublished retrospective review of my patient charts focused on AAD combinations (which does not consider β-blockers, calcium channel–blockers, or digitalis as part of the AAD combination), I identified several highly symptomatic AF patients who failed multiple AADs for inefficacy, including amiodarone, and in whom I tried AAD combinations—most occurring in the era before ablation therapy. Records were not included if a patient had had a bradycardic or proarrhythmic event on single AAD therapy or a clinical or electrocardiographic (ECG) contraindication to either agent to be tried. The efficacy of each combination was considered to be clinically significant if there was a reduction in AF frequency and duration to the level of the patient’s satisfaction. Considering results of the number of patients improved out of the number of patients tried, combinations that were successful included amiodarone + propafenone: nine of 12 patients (0.75%); amiodarone + disopyramide: four of five patients (80%) (mainly for vagal-induced AF characteristics); amiodarone + ranolazine: three of three patients (100%); dronedarone + ranolazine: nine of 10 patients (90%); and sotalol + propafenone: two of two patients (100%). No proarrhythmia or untoward bradyarrhythmia occurred in any patient. In each case, the lowest dose of each agent used in combination was the lowest starting dose typical for either agent separately. Thus, as a general observation, AAD-C approaches may be successful for AF when single agents fail and my experience using combinations of AADs with different mechanisms of action suggests the potential even for enhanced efficacy. Similar enhanced efficacy for AF has been reported in Asia with bepridil combined with either aprindine or a class Ic AAD.^25,26^ Importantly, since amiodarone appears to interact with almost every drug with which it has been tried and in a manner that increases the achieved concentrations of the concurrent agent, whenever I use a second AAD in combination with amiodarone, I always start with the lowest dose possible and increment the dose, if needed, very slowly, using electrocardiography, serum levels if available, and the patient’s responses as a guide. I also take this approach if the anticipated pharmacodynamic actions can produce untoward effects, such as bradycardia, that are already prominent with either drug alone.

In parallel, AADs may be effective but fail due to intolerance. I have also combined AADs, both at lower doses, to offset adverse effects when intolerance was the limitation to single-drug therapy. For example, per my patient chart review, several years ago, I used class I AADs in combination in nine highly symptomatic AF patients whose AF was suppressed with quinidine or disopyramide but in whom gastrointestinal (GI) intolerance was present with each—that is, diarrhea on quinidine and constipation on disopyramide, respectively. For each combination, a half-dose of each drug was used. In eight of the nine patients (89%), the combination achieved successful control of AF but without any GI intolerance. In more recent years, I have used the combination of dronedarone and ranolazine in patients where each drug alone was effective but where dronedarone caused diarrhea and ranolazine caused constipation. This strategy was, in part, stimulated by the results of the Study to Evaluate the Effect of Ranolazine and Dronedarone When Given Alone and in Combination in Patients with Paroxysmal AF (HARMONY).^27^ In each case, when the above combinations were used, I incorporated a reduced dose of ranolazine as compared with that used in its trial as monotherapy. However, as dronedarone is only available commercially in one dose (i.e., 400 mg twice daily), it was continued at this dose despite the fact that, in HARMONY, the combinations employed used reduced doses of dronedarone in conjunction with submaximally dosed ranolazine. Thus, for example, in my patients, after ranolazine was effective but not tolerated at 1 g twice daily and dronedarone was effective but not tolerated at 400 mg twice daily, the combination used with success was ranolazine 500 mg plus dronedarone 400 mg twice daily. The above experience is yet another example of the value of considering low-dose AADs.

## Concluding thoughts

The major impetus to using a low dose of any drug, including AADs, is to minimize patient risk and drug intolerance. In general, evidence-based doses such as those resulting from pivotal trials, detailed in PIs, or found in the medical literature are to be considered; in particular, PIs provide dosing data and ranges. Initiating with the lowest dose proven effective may hold especially true for amiodarone, following appropriate loading, given its almost innumerable toxicities and drug interactions. Using low doses of AADs in combination, especially if the drugs have differing electrophysiologic actions, may afford a degree of efficacy in some patients where each drug fails as a single agent while simultaneously reducing the risk of side effects that more typically occur at higher doses, providing another rationale for low-dose selection. Certainly, however, adverse effects that are idiopathic will not be avoided by adopting combination therapy. Similarly, even if individual AADs are effective, they may not be tolerated. In such cases, the use of combined AADs, each at a reduced dose, may provide both improved tolerance and efficacy.
